# Examining the photo catalytic potency of annealed and un-annealed ZnO and nickel doped ZnO for degradation of organic pollutants in waste waters

**DOI:** 10.1038/s41598-024-60258-5

**Published:** 2024-09-18

**Authors:** Fatima Gull, Rehana Riaz, Komal Ansari, Haleema Atiq

**Affiliations:** Department of Physics, Faculty of Sciences, International Islamic University, Islamabad, Pakistan

**Keywords:** Nanoscale materials, Nanoscience and technology

## Abstract

Water scarcity and pollution has increased the need for innovative and effective waste water treatment methods. The presented study aims to tackle this difficulty by synthesizing zinc oxide (ZnO) and nickel (Ni) doped ZnO to improve their photo catalytic capacity. This study examines wastewater treatment and organic pollutant breakdown using nanotechnology. The annealing increases photo catalytic activity by 65%, thereby enhancing efficiency. XRD shows that annealing decreased the average crystal size of pure ZnO and nickel doped ZnO (Ni:ZnO) i.e., for pure ZnO average crystal size is decreased from 23.90 to 20.90 nm and for Ni:ZnO, 34.39–28.65 nm. SEM shows that un annealed samples have agglomerates, while annealed samples are quasi-spherical. Using diffuse reflectance spectroscopy (DRS), the study examines how annealing affects optical band gap. Annealed Ni:ZnO has a band gap of 3.09 eV, which is smaller as compared to un annealed Ni:ZnO (3.18 e V). Similarly, the decline in energy band gap is observed for pure ZnO too. This study highlights the significant capacity of Ni:ZnO, for un annealed and annealed synthesis, to effectively meet the urgent requirements for waste water treatment. The extensive research conducted in this work enhances our comprehension of photo catalytic materials and underscores its potential for practical implementation in addressing waste water-related environmental issues.

## Introduction

The progression of technology and industrialization has undoubtedly enhanced the quality of human life but upset the ecosystem, especially sewage imbalances^1^. Water, being a universal soluble liquid and possessing the highest capacity to decompose substances among all liquids on the planet, is highly vulnerable to contamination^2^. Water quality will emerge as a substantial challenge for societies in the twenty-first century, considering that one in every nine individuals worldwide obtains their drinking water from untreated and potentially hazardous sources. Moreover, 90% of sewage discharged into untreated aquatic environments in developing countries is responsible for the daily discharge of 2 million tons of effluents. An annual industrial discharge of 300–400 mega tons of waste into water, a problem that is further compounded by agricultural and municipal activities, presents a global threat to biodiversity^3^. The issue of water pollution is a significant concern that poses a considerable threat to human health. In fact, the number of deaths resulting from water pollution surpasses the combined fatalities caused by war and violence each year, amounting to approximately 1.8 million deaths annually^4^. With less than 1 percent of the Earth's freshwater being readily accessible, the limited availability of drinkable water sources exacerbates the crisis. However, if no action is taken, it is anticipated that challenges will intensify, leading to a projected 33% rise in global freshwater demand by the year 2050^2^. Thus, efficient waste water treatment is necessary to prevent disease transmission and ecological harm from untreated waste water discharge^5^.

A significant portion of water pollution is attributed to organic contaminants found in sewage, industrial, and agricultural waste^6^. The presence of these substances can lead to adverse health effects in humans and other living organisms, as many of them have the potential to cause cancer and genetic mutations^7^. The majority of conventional techniques are unable to fully remove organic contaminants from waste water due to their low efficiency and lack of environmental compatibility^8^. Conversely, advanced oxidation processes (AOPs), including photolysis, UV (ultra-violet) radiation, photo catalysis, sonolysis, radiolysis, and ozonation^9–14^, have been proven effective in the elimination of organic contaminants from water sources. These processes enable the decomposition of water into carbon dioxide (CO_2_) and water (H_2_O) through oxidation and reduction reactions, thereby mitigating potential harm to the environment^15–17^. The current study is specifically centered on the topic of photo catalysis, mostly recognized for its environmentally friendly nature, high efficiency, low cost, chemical stability, and non-toxic properties^18–23^. Furthermore, organic compounds, including hazardous pollutants have the ability to undergo mineralization and complete decomposition when exposed to appropriate light through the process of photo catalysis^24–26^. This phenomenon can be done by utilizing many semiconducting materials i.e. ceric oxide (CeO_2)_, magnesium oxide (MgO), zinc sulfide (ZnS), titanium oxide (TiO_2_) and ZnO, as most of these materials limit their photo catalytic activity to UV region e.g. in comparison with TiO_2_, ZnO exhibits elevated surface reactivity due to greater reactive sites making it an adept photo catalyst under visible light^27,28^.

Zinc oxide’s direct band gap in the near-UV region, strong oxidation ability, efficient photo catalytic property, and greater free-exciton binding energy make it a leading candidate for sustainable environmental management systems^29–31^. However, zinc oxide's photo catalytic activity faces challenges like electron–hole aggregation, limited response to visible light, and susceptibility to photo-corrosion. Researchers address these issues by employing nano-junction systems or doping with elements and adjusting light absorption through metal addition or compositing with other semiconductors^32–35^. Firstly a study by Türkyılmaz et al.^36^ confirms that ZnO exhibited the highest efficiency in degrading tartrazine (azo dye) with Ni in comparison with doping of manganese (Mn), iron (Fe), and silver (Ag) at the same doping concentration. Also from literature study, it has been observed that Ni show enhanced degradation of methylene blue (organic pollutant) due to large band gap under visible light irradiation^37^. Furthermore, it has been observed that there is a direct correlation between the concentration of nickel in ZnO and the efficiency of degradation^38^.

Our research encompasses the comparative study of annealed and un-annealed synthesization of pure ZnO and nickel doped ZnO with 3% concentration of nickel in ZnO as investigated by Xu et al.^39^ that highest photo catalytic activity occurred for 3% nickel doped ZnO, moreover, methylene blue is utilized instead of sodium ligno sulfonate to investigate the percentage degradation at 800 °C for waste water treatments.

## Experimental section

### Materials

For the synthesis of ZnO and Ni:ZnO, zinc chloride (ZnCl_2_) and nickel chloride (NiCl_2_) were employed as precursor agents. As a solvent, distilled water (H_2_O) was utilized. Gel formation was facilitated by the utilization of sodium hydroxide (NaOH). Isopropanol alcohol (C_3_H_8_O) and nitric acid (HNO_3_) were employed to aid in the chemical synthesis. All of these materials were purchased from company Sigma Aldrish. To evaluate the photo degradation capability of the synthesized nanostructure, methylene blue (MB) ([7-(Dimethyl- amino) phenothiazin-3-ylidene]-dimethyla- zanium chloride) was employed as a pollutant.

### Synthesis

In this study, sol gel is adopted as the synthesis technique as it has been employed extensively in photo catalysis to achieve higher degradation efficiency^40,41^. Therefore, using the sol–gel technique nickel-doped ZnO having concentration (x (wt%) = 0%, 3%) was produced. A solution of sol can be obtained by combining a 2 M (molar) solution of NaOH with 1 M solution of ZnCl_2_ and agitating continuously at 80 °C for 60 min using a magnetic stirrer. As diluting solvents, five droplets of HNO_3_ and C_3_H_8_O were added to the prepared solution. By combining distilled water with 3% NiCl_2_, an additional solution of Ni^+2^ ions was produced. An additional ZnO solution was supplemented drop by drop with this NiCl_2_.6H_2_O solution. After 25 min of agitating and heating, the two solutions were centrifuged at 2500 rpm for an additional 35 min. Throughout the synthesis, the subsequent reaction occurred:

1 $$ {\text{ZnCl}}_{{2{ }}} + 2{\text{NaOH }} \to {\text{Zn}}({\text{OH}})_{2} { } + 2{\text{NaCl}} $$

2 $$ {\text{Zn}}({\text{OH}})^{2} { } + { }2{\text{NaCl }} \to {\text{ZnO }} + {\text{ H}}_{2} {\text{O }} + { }2{\text{NaCl}} $$

For nickel doped ZnO,

3 $$ {\text{Zn}}({\text{OH}})^{2} + {\text{NiCl}}_{2} .6{\text{H}}_{2} {\text{O}} \to {\text{Ni}}:{\text{ZnO }} + {\text{Cl}}_{2} + 7{\text{H}}_{2} {\text{O}} $$

The solutions were allowed to sediment for thirty minutes and were rinsed three times to remove any contaminants. The dip-coating approach was used to deposit thin films of ZnO and nickel doped ZnO onto a glass substrate. A direct correlation is confirmed by a prior research between heightened annealing temperatures and elevated crystallinity^42^. Our XRD analysis also confirmed that the annealed samples exhibited a more pronounced crystalline structure compared to their un annealed counterparts. Thus, for 60 min the thin films that had been made for each sample were placed in the furnace and annealed at 800 °C with the chosen temperature based on our laboratory’s capabilities.

### Photo catalysis

The experiment was conducted using an organic dye pollution called methylene blue (C_16_H_18_CIN_3_S). A solution containing 10 ppm of MB was prepared, and 0.005 g of ZnO and Ni:ZnO were added to the solution. In order to achieve absorption/desorption equilibrium, the solution was agitated and stored in an opaque container for a duration of 60 min. A 100 W tungsten lamp was utilized as a radiation source. The bulb and the solution was separated by a space of 10 cm. The degradation was assessed following a 60 min period. The maximal absorbance intensity of methylene blue using UV–Visible spectrophotometry was observed at a wavelength of 605 nm as the irradiation was done by visible light source (tungsten lamp) depicted in the Fig. 5. The degradation efficiency of methylene blue was assessed for all samples using the following equation:

4 $$ {\text{Degradation}}\;{\text{rate}}\;\% = \frac{{{\text{A}}_{{\text{o}}} - {\text{A}}_{1} }}{{{\text{A}}_{{\text{o}}} }}{ } \times 100 = \frac{{{\text{C}}_{{\text{o}}} - {\text{C}}_{1} }}{{{\text{C}}_{{\text{o}}} }}{ } \times 100{ } $$

A_o_ represents the absorbance of un doped MB, while A_1_ represents the absorbance of MB containing annealed Ni:ZnO.

### Mechanism of photo catalysis

From the recent researches it is evident that photo catalysis is considered as an environmental friendly technique to remove pollutants from the waste waters^43,44^. Photo catalysis take places by generating harmless hydroxyl and superoxide radicals whenever the photo catalyst is exposed to a light source (UV light, Visible light, Sunlight), electron from valence band (VB) moves to conduction band (CB) and a hole is generated in the VB. This electron combines with oxygen (O_2_) creating anion superoxide radicals (^·^O_2_^-^), the reaction further precedes by creating free radicals (·OH) when hole (h^+^) reacts with water molecules (H_2_O). This reactivity ends with the production of nontoxic H_2_O_2_ from cation/anion reaction and the degraded pollutants. The pollutant degraded products are in the form of SO_4_ and NO_3_^45^. The reactions taking place are,

5 $$ Photo\;catayst +{\text{h}{\upnu}} \to {\text{e}}^{ - } + {\text{h}}^{ + } $$

6 $$ {\text{e}}^{ - } + {\text{O}}_{2 } \to ^{ \cdot } {\text{O}}_{2}^{ - } $$

7 $$ {\text{h}}^{ + } + {\text{H}}_{2} {\text{O}} \to^{ \cdot } {\text{OH}} + {\text{H}}^{ + } $$

8 $$ {\text{h}}^{ + } + {\text{OH}}^{ - } \to^{ \cdot } {\text{OH}} $$

9 $$ ^{ \cdot } \text{O}_{2}^{ - } + \text{H}^{ + } \to^{ \cdot } \text{OOH} $$

10 $$ 2^{ \cdot } {\text{OOH}}^{ + } \to {\text{O}}_{2} + {\text{H}}_{2} {\text{O}}_{2} $$

11 $$ {\text{H}}_{2} {\text{O}}_{2} +^{ \cdot } {\text{O}}_{2} \to ^{ \cdot } {\text{OH}} + {\text{OH}}^{ - } + {\text{O}}_{2} $$

12 $$ {\text{H}}_{2} {\text{O}}_{2} + {\text{h}{\upnu}} \to 2^{ \cdot } {\text{OH}} $$

13 $$ Pollutant + \left( {^{ \cdot } \text{OH}, \text{h}^{ + } ,^{ \cdot } \text{OOH}/\text{O}_{2} } \right) \to degraded\;pollutant $$

Photo catalysis is known to be popular for degradation of pollutants because of its widespread scalability and environmental remediation^46,47^. Researches have been modified since 2016 to enhance the efficiency of photo catalytic activity i.e. doping photo catalysts with hetero atoms (S, N, P, O)^48^ and transition metals (Ni, Fe, Ti) or alkali metals (Li, Na, K) is proved to have a long term stability and reusability^49^. In terms of economical feasibility, photo catalysis outstands because of its low-cost in large scale applicability to treat contaminated water^50^.

### Characterization

Using an X-ray diffractometer (XPERT-3 with Cu (copper) K_α_ radiations), structural analysis was performed on as-prepared samples of thin films deposited on a glass substrate. The apparatus was equipped with a wavelength of 1.540589 Å and operated at a voltage of 40 kV and an applied current of 40 mA. The morphology of nanostructures was analyzed using scanning electron microscopy (JSM-6490) with an accelerating voltage of 20 kV. The photos were analyzed at a resolution of 1 µm. The spectrophotometer (Schimadzu 2700-UV) was used to evaluate the optical characteristics and photo catalytic activity.

## Results and discussions

### X-ray diffraction (XRD)

**Figure 1:**
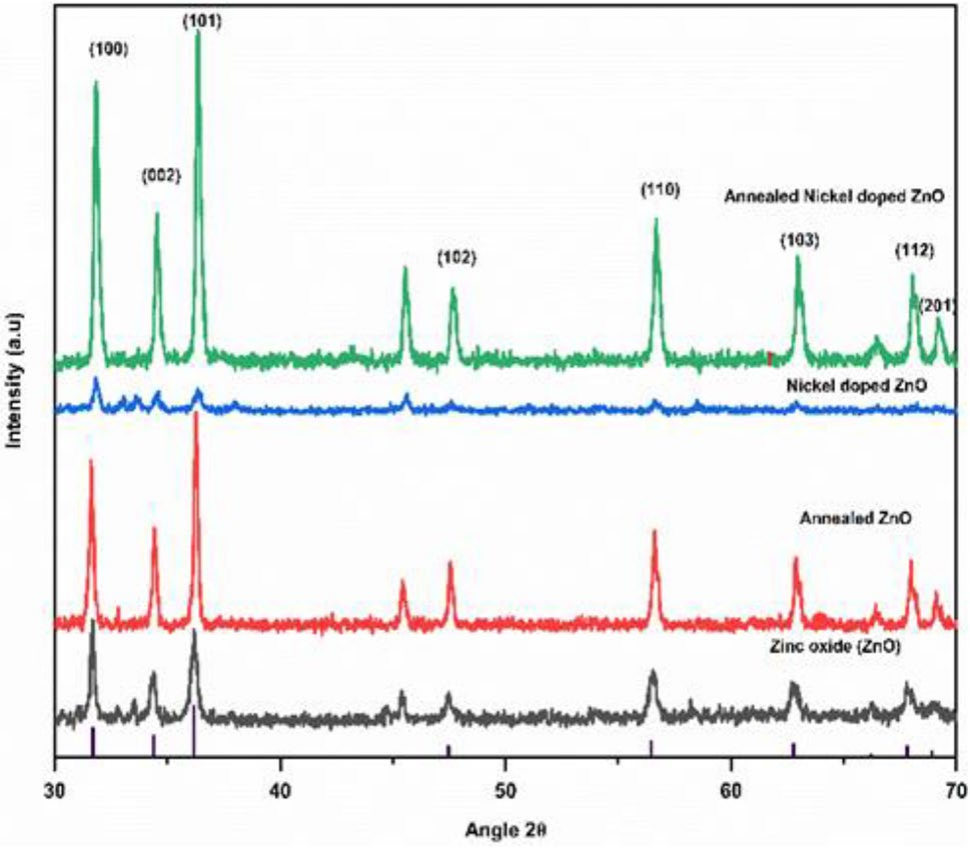
XRD spectrums of all samples.

X-ray diffraction (XRD) analysis was conducted in order to gain a deeper comprehension of the crystal structure and phase purity of ZnO, annealed ZnO, nickel-doped ZnO, and annealed nickel-doped ZnO. Figure 1 presents the X-ray diffraction (XRD) diffractogram patterns of the un annealed and samples subjected to annealing, at a temperature of 800 °C. The patterns revealed consistent alignment of all peaks to the standard data (JCPDS, 36-1451) within the P6_3_mc space group of hexagonal ZnO wurtzite. The findings indicate a strong correlation between the diffraction patterns and the indexing of the hexagonal wurtzite structure of ZnO with respect to the planes (100), (002), (101), (102), (110), (103), (112), (201). Nevertheless, it is apparent that not all precursor elements underwent complete decomposition, as indicated by the existence of a secondary peak at a 45° inclination that corresponded to the structure of zinc hydroxide. Similar peak of impurity was observed in the diffraction pattern of previous literatures^51^.

Nanostructures of all samples have (101) orientation as the peak exhibits the highest intensity and indicates a favored growth plane The diffractogram pattern of Ni:ZnO do not exhibit any discernible peak that suggests the presence of nickel or its derivatives, hence confirming the purity of nickel-doped zinc oxide. Nevertheless, notable alterations were observed in the majority of the peaks correlating our results with recent researches^52^. The lack of secondary phases, such as clusters or oxides, suggests that nickel has been integrated into the lattice as a substitutional atom. The average crystal sizes of synthesized nanostructures were calculated by a Debye Scherrer formula. Analysis revealed that nickel incorporation in zinc slightly increases the crystal size i.e., for un annealed zinc oxide the approximate particle size is 23.90 nm which reduces to 34.39 nm when we doped it with nickel. Similar results are observed for annealed samples of ZnO and Ni:ZnO i.e., size shifted from 20.90 to 28.65 nm. This is explained by the fact that when nickel is incorporated in zinc oxide it hinders ZnO crystallization as the ionic radii of nickel i.e., 0.68 Å is smaller than that of zinc oxide i.e., 0.74 Å, thus ultimately decreasing the intensity of the peaks^53^.

Moreover crystallite size and intensities of the diffraction peaks exhibited an upward trend as the samples were annealed at 800 °C, indicating enhanced crystallinity compared to un annealed one’s^54–56^. This observation suggests that a higher annealing temperature supplies ample energy for the crystallization process to occur, resulting in the proper orientation of crystals at equilibrium sites and leading to an increase in intensity.

Several crystallographic parameters are presented in Table 1:

**Table 1:** Crystallographic parameters of all the samples.

Samples	2θ (101)	D (nm)	Dislocation density	Lattice parameter (a)	Lattice parameter (c)
ZnO	36.18	23.90695172	1.750 × 10^–3^	2.8645	4.96
Ni:ZnO	36.26	34.39713956	8.452 × 10^–4^	2.8584	4.95
Annealed ZnO	36.34	20.90737557	2.287 × 10^–3^	2.8523	4.94
Annealed Ni:ZnO	36.35	28.65695101	1.220 × 10^–3^	2.8515	4.93

The lattice constants (a and c) of ZnO are determined from the following equations:

14 $$ a = \frac{\lambda }{\sqrt 3 \sin \theta } $$

and,

15 $$ c = \frac{\lambda }{\sin \theta } $$

Additionally, the dislocation density (δ) represents the defects in the crystal growth due to the lattice mismatch between substrate and nanostructure deposition and impurities. The (δ) value is inversely proportional to the square of the crystal size as,

16 $$ \delta = \frac{1}{{D^{2} }} $$

Also there observed a slight decrease in the value of lattice parameters both by annealing and Ni doping. ZnO nanostructures may typically have a number of defects such as oxygen vacancies, lattice disorders, etc. The annealing process eliminates these defects from the structures, resulting in a more compact lattice. Additionally, the lattice relaxation caused by suspended bonds must be taken into account. An electrostatic attraction is generated between the oxygen ions present in the air and the suspended bonds on the zinc oxide surface, resulting in a minor contraction of the lattice^57,58^. Nevertheless, the addition of nickel results in a more pronounced contraction of the lattice due to the smaller size of nickel ions in comparison to zinc ions^59^.

### Scanning electron microscopy (SEM)

**Figure 2:**
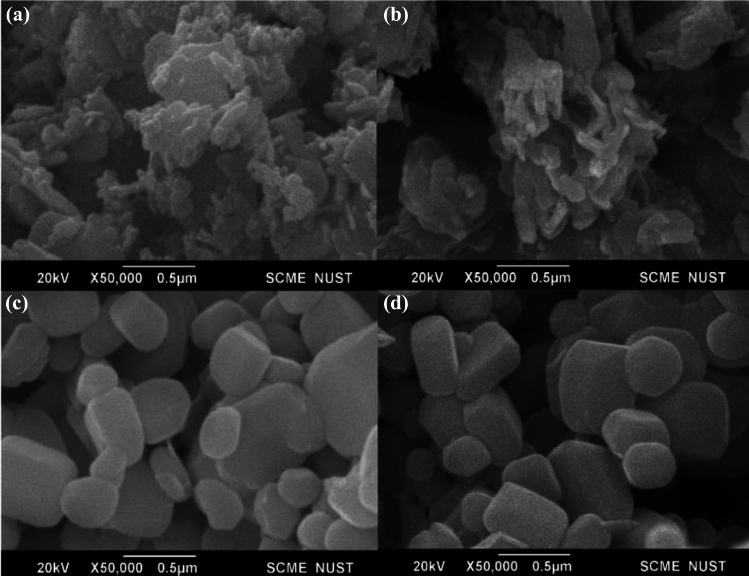
SEM images of (**a**) ZnO, (**b**) Ni:ZnO, (**c**) annealed ZnO, (**d**) annealed Ni:ZnO.

The scanning electron microscope (SEM) images of four different samples are displayed in Fig. 2a, b, c, and d. These samples include pure ZnO, annealed ZnO, nickel-doped ZnO, and annealed nickel-doped ZnO. The morphology of both the un annealed samples consists of nano rods with a diameter of 23.90 nm for ZnO and 20.90 nm for Ni:ZnO. These particles are not individually separated, but instead exist in the form of agglomerates. Upon annealing, the morphology undergoes a transformation into nano spheres characterized by clearly defined grain boundaries. The alteration in shape can be elucidated by the occurrence of Ostwald ripening. Initially, the particles were grouped together, owing to their elevated surface energy, they tend to enlarge into quasi-spherical formations by annealing at a temperature of 800 °C. This study corresponds with prior research by Pudukudy et al.^60^ confirming that with the increase in temperature the morphology is modified from ZnO nano particles into quasi-spheres. The correlation between particle aggregation (in un annealed samples) and photo catalytic activity in our study aligns with past research indicating that aggregation affects material optical properties and, consequently, photo catalytic performance^61^. Moreover, findings by Deng et al. reveals that the spherical morphology, post annealing enhances photo catalytic degradation as compared to commercial zinc oxide^62^.

### Diffuse reflectance spectroscopy (DRS)

**Figure 3:**
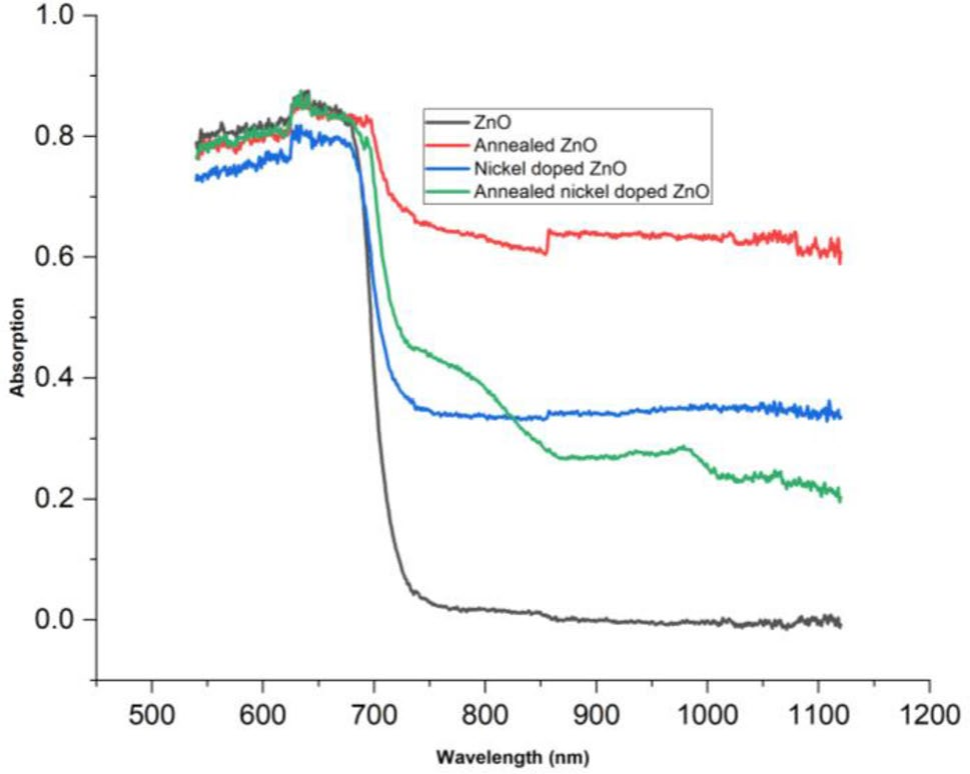
Absorption spectra of all samples.

**Figure 4:**
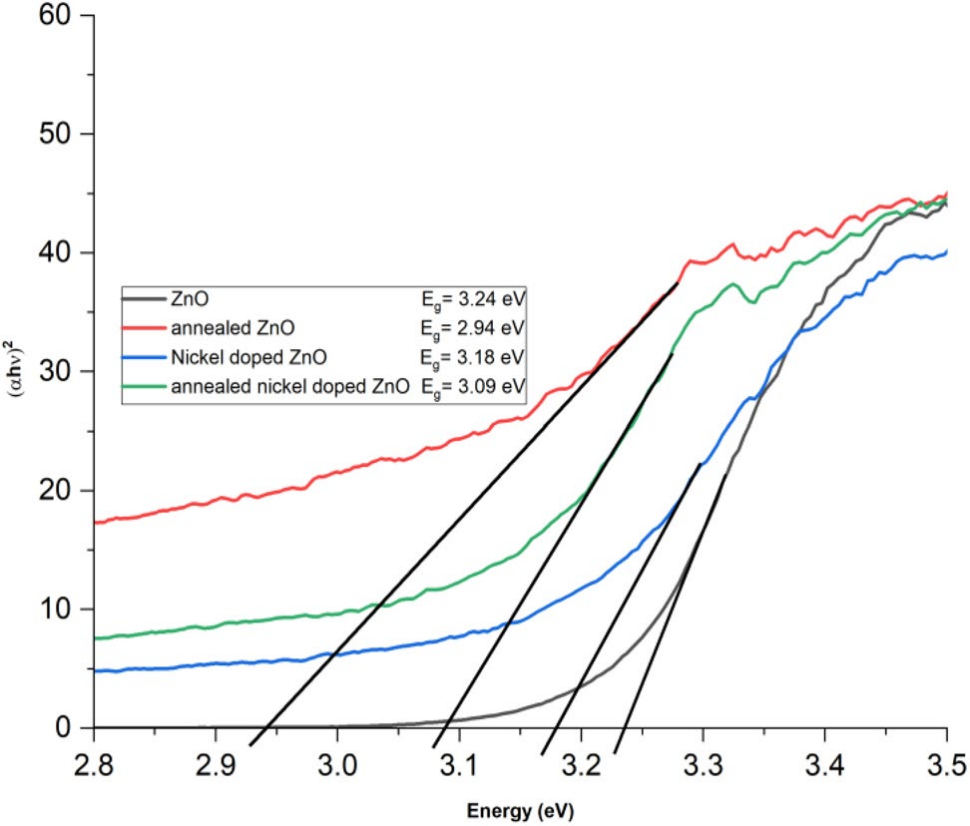
Optical band gaps of all samples.

Figure 3 illustrates the optical absorption spectra of ZnO nano rods, annealed ZnO nano sheres, nickel-doped ZnO nano rods, and annealed nickel-doped ZnO nano spheres at room temperature. The optical band gaps of all the samples at room temperature are graphically represented in Fig. 4 via Tauc’s plot. Figure 4 illustrates the optical band gap through the additional depiction of the straight line. The maximum absorption peaks of these samples are observed at wavelengths of 316, 318, 310, and 313 nm. We have,

17 $$ {{\upalpha} \text{h}{\upnu}}  \propto (\text{h}{\upnu}  - {\text{E}}_{{\text{g}}} )^{{\text{n}}}  $$

where

18 $$ {\text{h}{\upnu}} = { }\frac{{1240{ }\left( {{\text{eV }} \times {\text{nm}}} \right)}}{{{\lambda  }\left( {{\text{nm}}} \right)}} $$

and,

19 $$ {\upalpha  } = { }\frac{{2.302{\text{A}}}}{{\text{d}}} $$

α represents the absorption coefficient, A represents the absorbance, and d represents the thickness taken as unity. To determine the direct optical band (allowed transition), a graph is generated plotting (αhv)^2^ against energy (hν). The oxygen vacancies and defects on the surface of ZnO resulted in a decrease in the optical band gap of ZnO from its typical value of 3.30 eV in bulk ZnO to 3.24 eV, leading to a red shift. The optical band gap of pure ZnO is decreased to 2.94 eV after annealing at a temperature of 800 °C. This reduction can be attributed to a decrease of the formation of defects^63^. The observed reduction in the band gap energy of ZnO due to annealing at higher temperature i.e. 800 °C can be ascribed to the enhanced crystallinity^64^.When nickel is incorporated, the optical band gap of pure ZnO decreases from 3.24 to 3.18 eV due to electronic transitions occurring towards the conduction band. The optical band gap decreased from 3.18 to 3.09 eV after annealing, indicating a red shift. This shift is caused due to the band electrons interacting (sp-d interaction) with the bounded d-electrons of the Ni^+2^ ions^65^. These results correlates with the results reported in^66^ where optical band gap decreases with Ni doping resulting into enhanced photo catalytic activity. From the previous literature, it has been found that doping ZnO with transition metals are effective for degradation of pollutants as they can cause decrease in band gap by absorbing more visible light thus imparting greater impurity levels^67–70^.

### Photo catalysis

**Figure 5:**
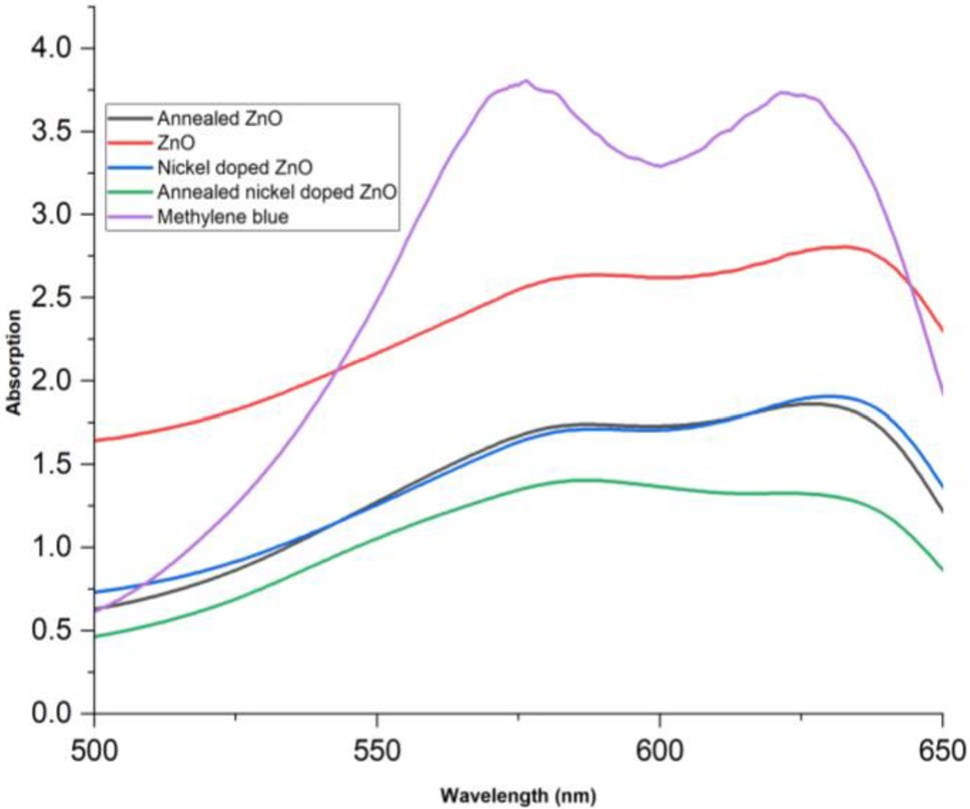
Absorption spectra for all samples.

**Figure 6:**
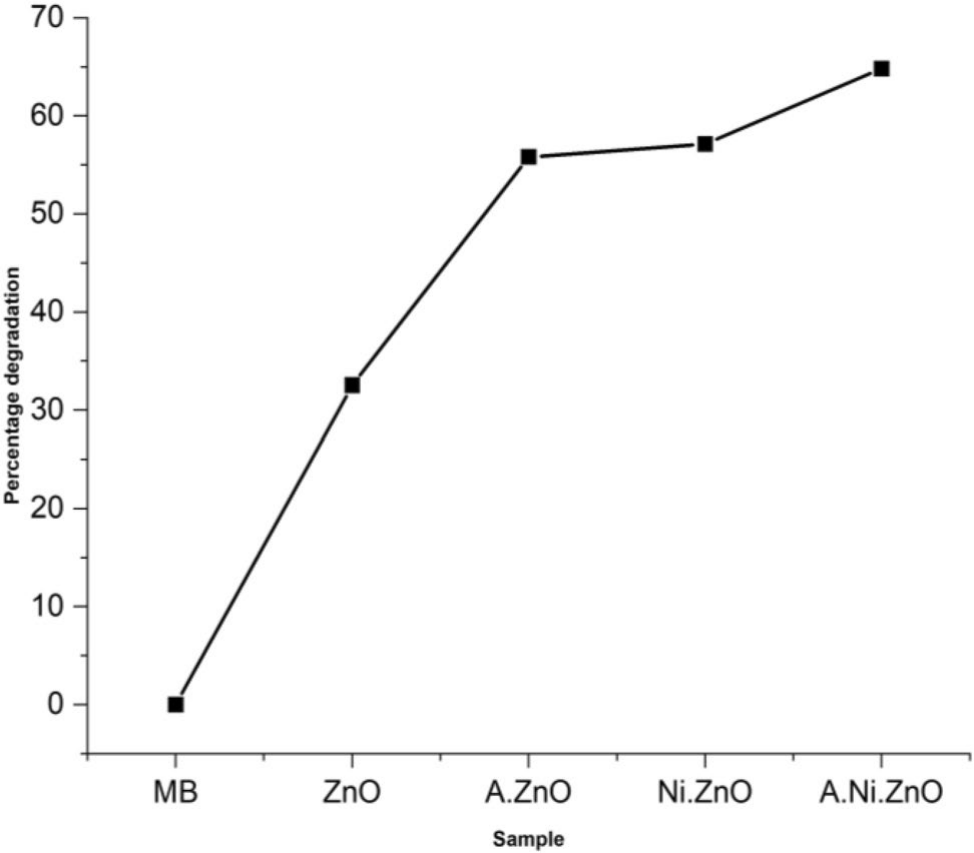
Percentage degradation of methylene blue with all samples.

Methylene blue (MB), a thiazine pollutant with extensive recognition and high carcinogenic potential, has been manufactured and employed in a wide range of industrial sectors for diverse purposes having applications in the coloring of cotton, wool, and fabrics, paper, hair^71^. Photo catalysis is employed to tackle the toxicity of MB as the provided Fig. 5 illustrates the absorption spectrum of methylene blue when exposed to various dopants and treated with light for a duration of 1 h. MB has its highest level of absorption at a wavelength of 605 nm, with a maximum absorbance peak of 3.8. However, when treated with annealed nickel doped ZnO, the absorbance is lowered to 1.3. The Fig. 6 illustrates that the photo catalytic activity of MB was enhanced by annealed nickel-doped ZnO. As, the annealed nickel doped ZnO catalyst exhibited the highest photo degradation efficiency of 65% after a duration of 60 min as compared to all the other samples (percent degradation for MB (0%), ZnO (32%), annealed ZnO (56%) and Ni:ZnO (57%). Thus, the non-annealed samples have exhibited a lower level of degradation in comparison to the annealed nickel doped ZnO. The presence of metal ions as a doping element in ZnO is responsible for the shift in fermi level towards a negative potential and the production of adequate hydroxyl group^72^. The increased photo catalytic activity is attributed to doping as it diminishes the possibility of electron and hole recombination, leading to improved charge separation in Ni-doped materials.

Moreover, the incorporation of Ni substitution serves to capture electrons, thereby reducing the likelihood of recombination of charges, which is a major impediment to the photo catalytic process^37^. Additionally, XRD analysis revealed that annealing Ni:ZnO increased the intensity of plane (101) and caused its dimension to vary between 20.90 and 28.65 nm as compared to annealed ZnO which corresponds to the research conducted by Abdel wahab et al. that increase in particle size increases the photo catalytic degradation^66^; consequently, in our research the enhanced crystallinity and surface-to-volume ratio of the synthesized nano spheres results in decreased band gap and increased photo catalytic activity. Thus altogether, the MB's photo catalytic activity is undoubtedly enhanced by each of these factors.

## Conclusion

Our research aims to enhance waste water treatment methods by enhancing the photo catalytic performance of zinc oxide through the process of nickel doping and precision annealing. This strategic adjustment results in a notable 65% improvement in photo catalytic activity, demonstrating an increase in efficacy. The X-ray diffraction (XRD) examination shows an improvement in crystallinity after annealing, while the scanning electron microscopy (SEM) reveals noticeable changes in the shape and structure of both pure ZnO and nickel-doped ZnO. The study of optical characteristics by diffuse reflectance spectroscopy (DRS) highlights the influence of annealing on band gaps, revealing that annealed nickel-doped ZnO demonstrate a smaller band gap. The research highlights the potential of nickel-doped ZnO, particularly after annealing at 800 °C, in efficiently tackling waste water treatment difficulties and providing vital insights into their practical uses in environmental remediation.

## Supplementary Information

Supplementary Information.

## Data Availability

All data generated or analyzed during this study are included in its Supplementary Information files.
