# Supplementary material for: Examining the photo catalytic potency of annealed and un-annealed ZnO and nickel doped ZnO for degradation of organic pollutants in waste waters
Source: Sci Rep. 2024 Sep 18;14:21828. doi: 10.1038/s41598-024-60258-5 (PMC11411114; doi:10.1038/s41598-024-60258-5)

Supplementary Fig. S1

Title

X-ray Diffraction (XRD) Spectrum of all the samples (ZnO, nickel doped ZnO, annealed ZnO and annealed nickel doped ZnO

— XRD spectrum of annealed nickel doped ZnO
—XRD spectrum of nickel doped ZnO
—XRD spectrum of annealed ZnO
—XRD spectrum of ZnO

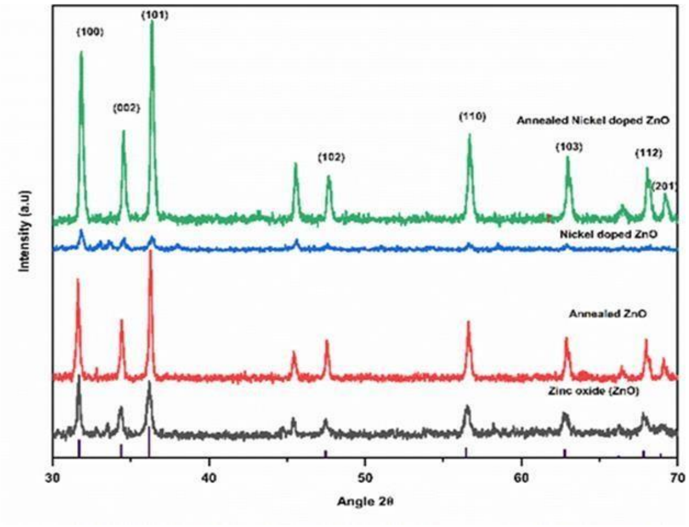

Supplementary Fig. S2

(a) **Title**

Scanning electron microscopy (SEM) images of ZnO

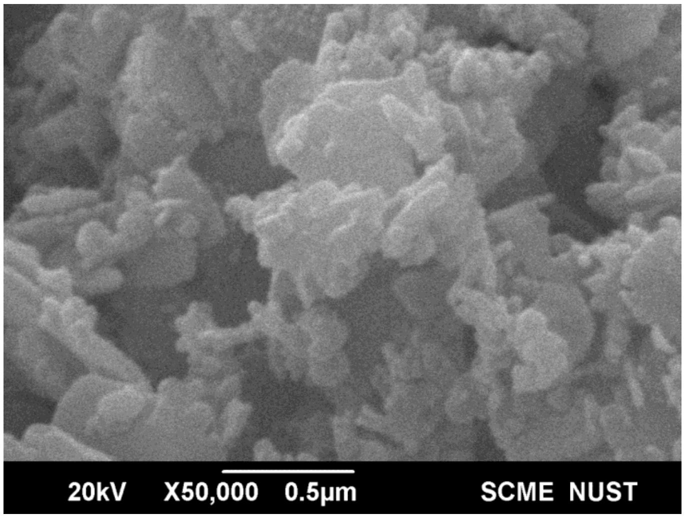

(b) **Title**

Scanning electron microscopy (SEM) images of nickel doped ZnO

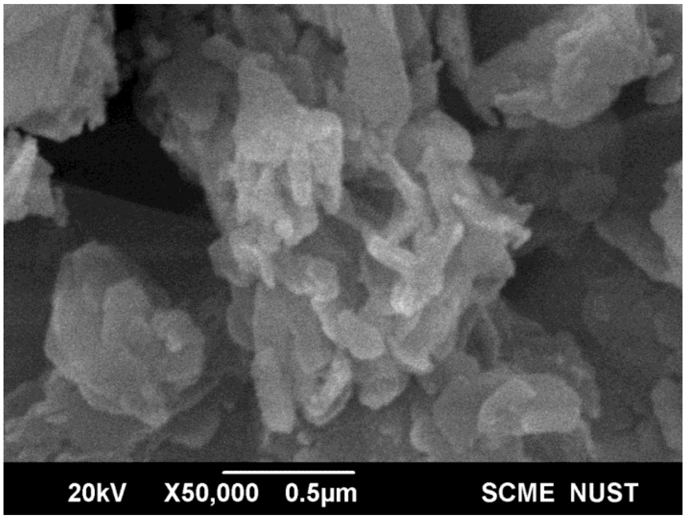

(c) **Title**

Scanning electron microscopy (SEM) images of annealed ZnO

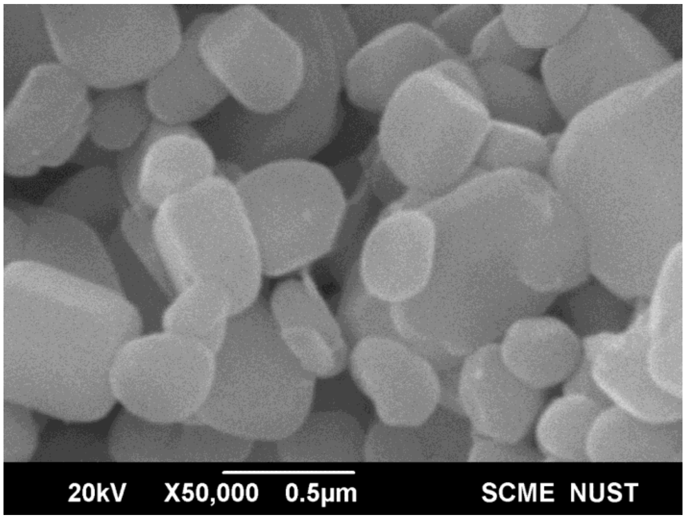

(d) **Title**

Scanning electron microscopy (SEM) images of annealed nickel doped ZnO

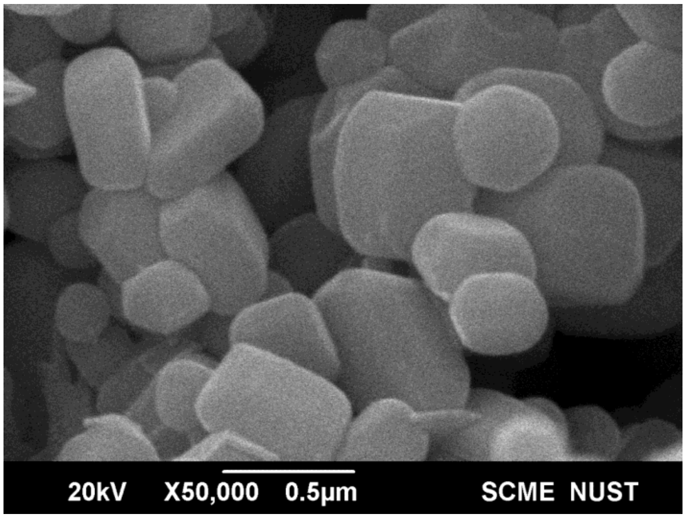

Supplementary Fig. S3

Title

Absorption spectrum of all the samples (ZnO, nickel doped ZnO, annealed ZnO and annealed nickel doped ZnO)

— Absorption spectrum of annealed nickel doped ZnO
— Absorption spectrum of nickel doped ZnO
— Absorption spectrum of annealed ZnO
— Absorption spectrum of ZnO

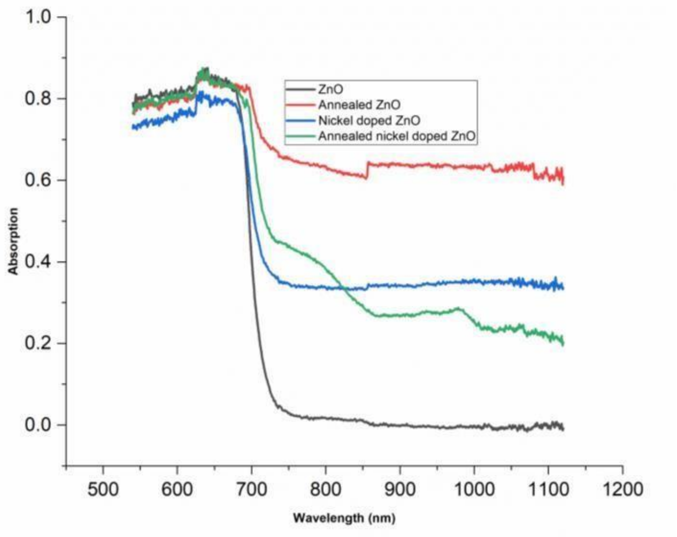

Supplementary Fig. S4

Title

Optical band gaps of all the samples (ZnO, nickel doped ZnO, annealed ZnO and annealed nickel doped ZnO) via taucs plot

— Taucs plot of annealed nickel doped ZnO E_g = 3.09 eV
— Taucs plot of nickel doped ZnO E_g = 3.18 eV
— Taucs plot of annealed ZnO E_g = 2.94 eV
— Taucs plot of ZnO E_g = 3.24 eV

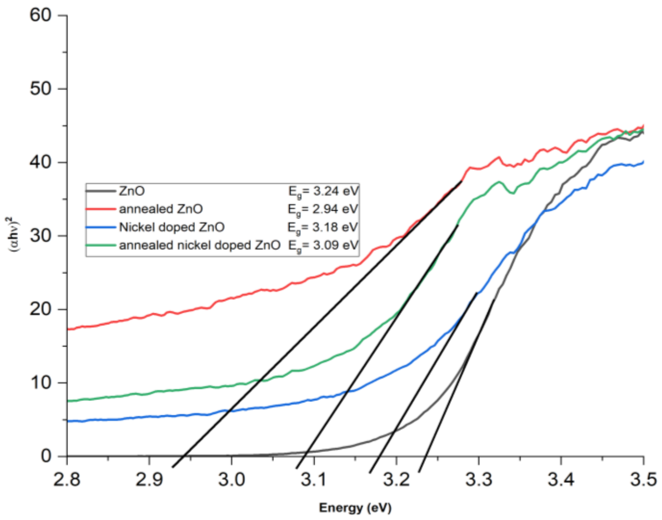

Supplementary Fig. S5

Title

Photo catalytic absorption spectrum of all the samples (ZnO, nickel doped ZnO, annealed ZnO and annealed nickel doped ZnO) after 60 minutes

— Absorption spectrum of annealed nickel doped ZnO
— Absorption spectrum of nickel doped ZnO
— Absorption spectrum of ZnO
— Absorption spectrum of annealed ZnO
— Absorption spectrum of methylene blue

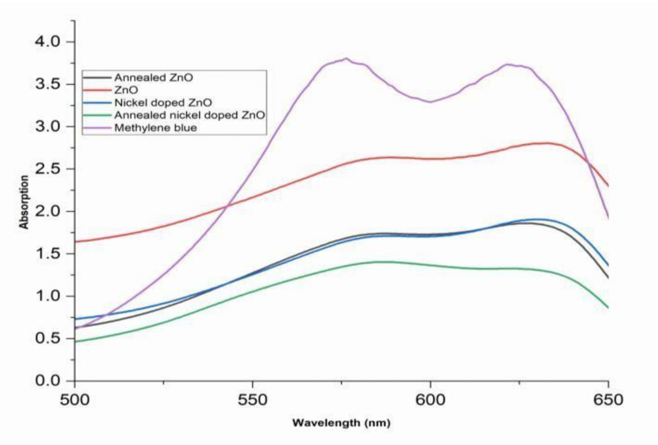

Supplementary Fig. S6

Title

Percentage photo degradation of methylene blue with all the samples (ZnO, nickel doped ZnO, annealed ZnO and annealed nickel doped ZnO)

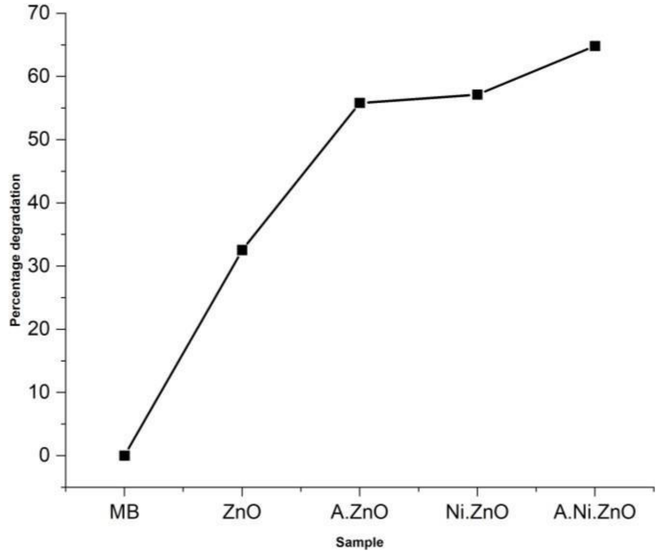

Supplement: Supplementary file 1 — Supplementary Information. [file 41598_2024_60258_MOESM1_ESM.pdf]
